# Supplementary material for: TERRA-LSD1 phase separation promotes R-loop formation for telomere maintenance in ALT cancer cells
Source: Nat Commun. 2024 Mar 9;15:2165. doi: 10.1038/s41467-024-46509-z (PMC10925046; doi:10.1038/s41467-024-46509-z)
Supplement: Supplementary file 1 — Supplementary Information [file 41467_2024_46509_MOESM1_ESM.pdf]

Supplementary Materials for

TERRA-LSD1 phase separation promotes R-loop formation for telomere maintenance in ALT cancer cells

Meng Xu *et al.*

* Corresponding author: huaiyinz@andrew.cmu.edu

This file includes:

Supplementary Figure 1 to 10
Supplementary Table 1 to 2

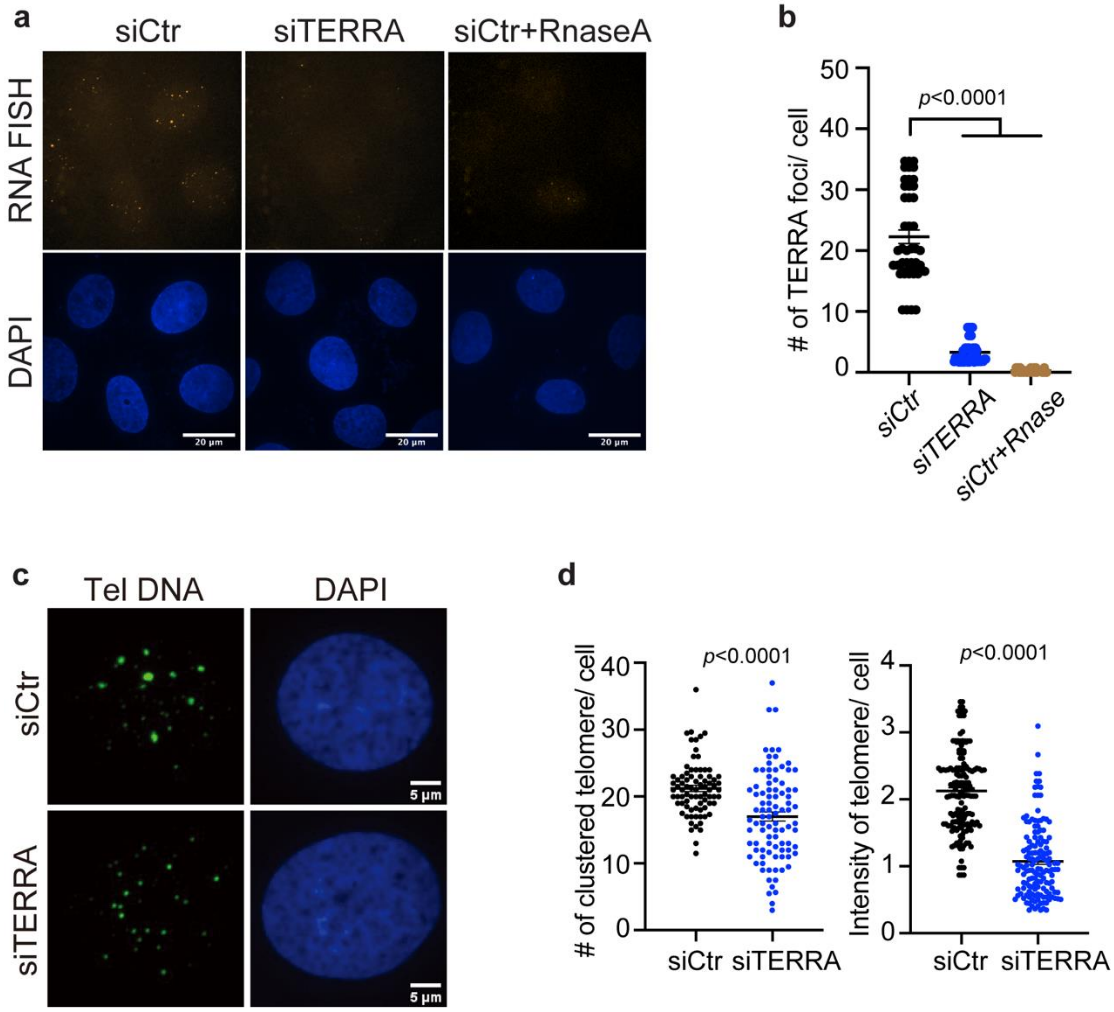

Supplementary Figure 1. High efficiency of TERRA knockdown results in decreased telomere clustering in U2OS cells. a Representative images and **b** quantification of TERRA foci (RNA FISH) after siTERRA or RNase A treatment in U2OS cells (n= two independent experiments, two-tailed unpaired *t* test). **c** Representative images of telomere DNA FISH and **d** quantification of telomere clustering (see Method) after siTERRA in U2OS cells synchronized in G2 (n= 161 cells examined for siCtr, n= 144 cells examined for siTERRA over three independent experiments, two-tailed unpaired *t* test). Error bars are mean ± SEM. Source data are provided as a Source Data file.

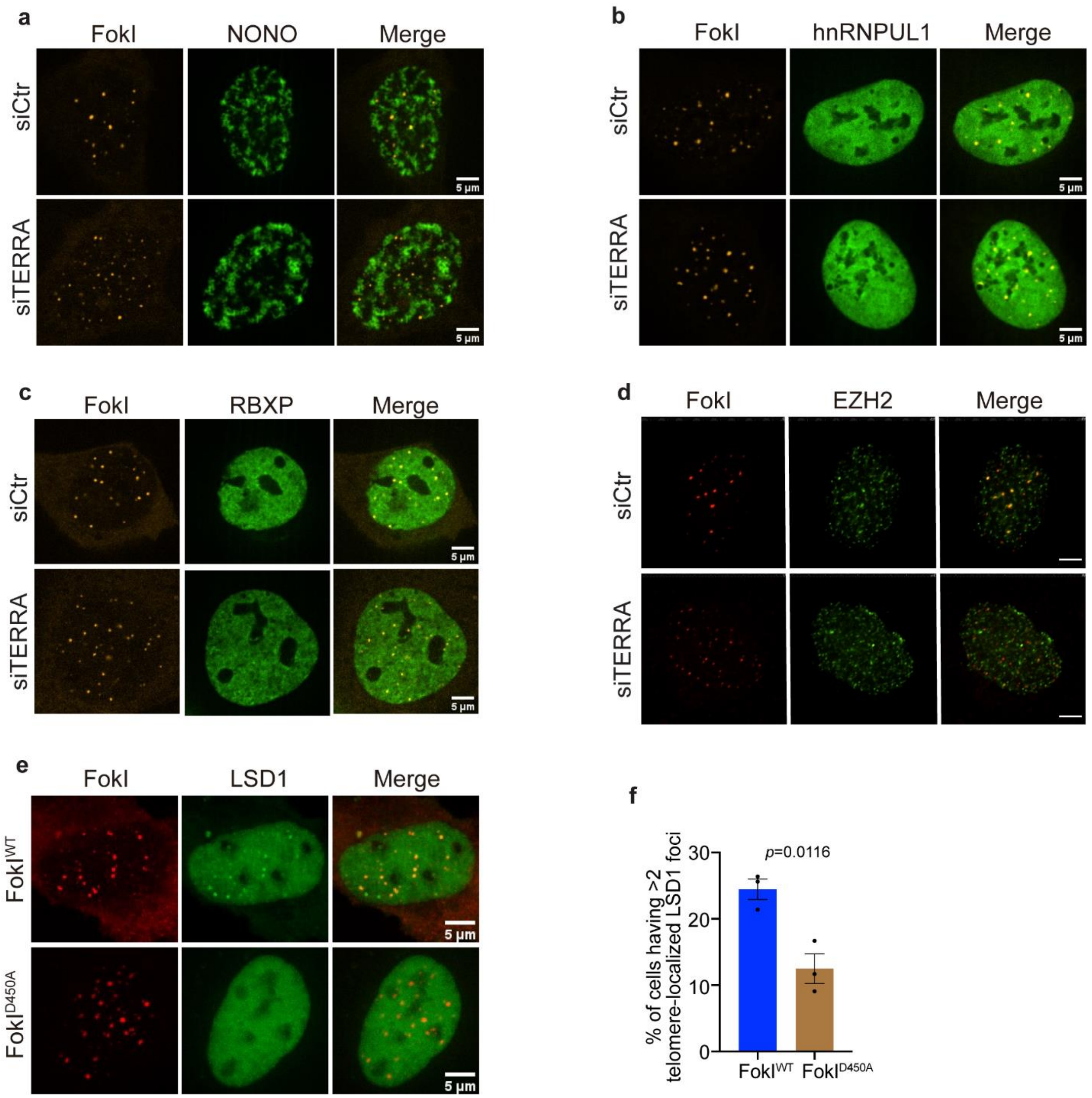

Supplementary Figure 2. Localization of RNA interacting proteins on telomeres. a-d Representative images of NONO **(a)**, hnRNPUL1 **(b)**, RBXP **(c)** and EZH2 **(d)** foci on telomeres after siTERRA in U2OS cells. Quantifications are in Fig. 1J. **e** Representative images and **f** quantification of LSD1 localization on telomeres after co-transfection with mCh-FokI^{WT} and catalytically dead mutant mCh-FokI^{D450A} (n= three independent experiments, two-tailed unpaired *t* test). Error bars are mean ± SEM. Source data are provided as a Source Data file.

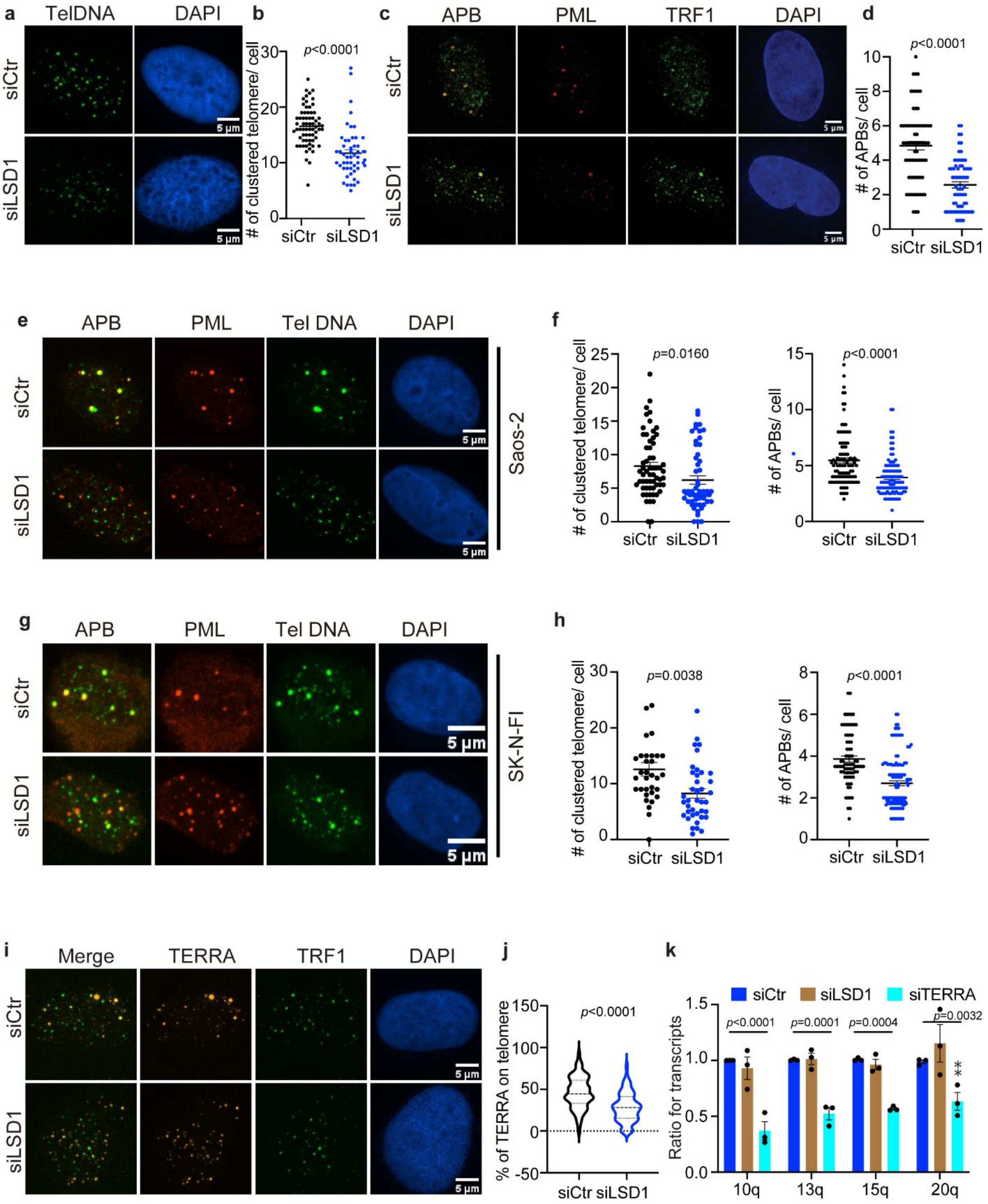
a
TelDNA
DAPI
siCtr
siLSD1
5 μm
b
p<0.0001
# of clustered telomere/ cell
siCtr
siLSD1
c
APB
PML
TRF1
DAPI
d
p<0.0001
# of APBs/ cell
e
APB
PML
Tel DNA
DAPI
Saos-2
f
p=0.0160
p<0.0001
g
SK-N-FI
h
p=0.0038
i
Merge
TERRA
TRF1
DAPI
j
p<0.0001
% of TERRA on telomere
k
siCtr
siLSD1
siTERRA
Ratio for transcripts
p<0.0001
p=0.0001
p=0.0004
p=0.0032
10q
13q
15q
20q

Supplementary Figure 3 Depletion of LSD1 causes ALT signature and reduced TERRA accumulation on telomere. a Representative images of telomere DNA FISH and **b** quantification of telomere clustering after siLSD1 in U2OS cells synchronized in G2 (n= 90 cells examined for siCtr, n= 108 cells examined for siLSD1 over two independent experiments, two-tailed unpaired *t* test). **c** Representative images and **d** quantification of APB bodies after siTERRA in U2OS cells synchronized in G2 (n= 71 cells examined for siCtr, n= 70 cells examined for siLSD1 over two independent experiments, two-tailed unpaired *t* test). Representative images and quantification of clustered telomere and APB on telomeres after siLSD1 in Saos-2 **e-f** (n= 104 cells examined for siCtr, n= 106 cells examined for siLSD1 over two independent experiments, two-tailed unpaired *t* test) and SK-N-FI **g-h** ALT+ cells (n= 79 cells examined for siCtr, n= 115 cells examined for siLSD1 over two independent experiments, two-tailed unpaired *t* test). **i** Representative images and **j** quantification of TERRA (RNA FISH) localization on telomeres after siLSD1 in G2 cells (n= 136 cells examined for siCtr, n= 127 cells examined for siLSD1 over two independent experiments, two-tailed unpaired *t* test). **k** TERRA levels from chromosome 10, 13, 15, 20 were quantitated by qRT-PCR, following transient knockdown of LSD1 and TERRA in U2OS for 48 hr (n= three independent experiments, one-way ANOVA). Error bars are mean ± SEM. N.S., not significant. Source data are provided as a Source Data file.

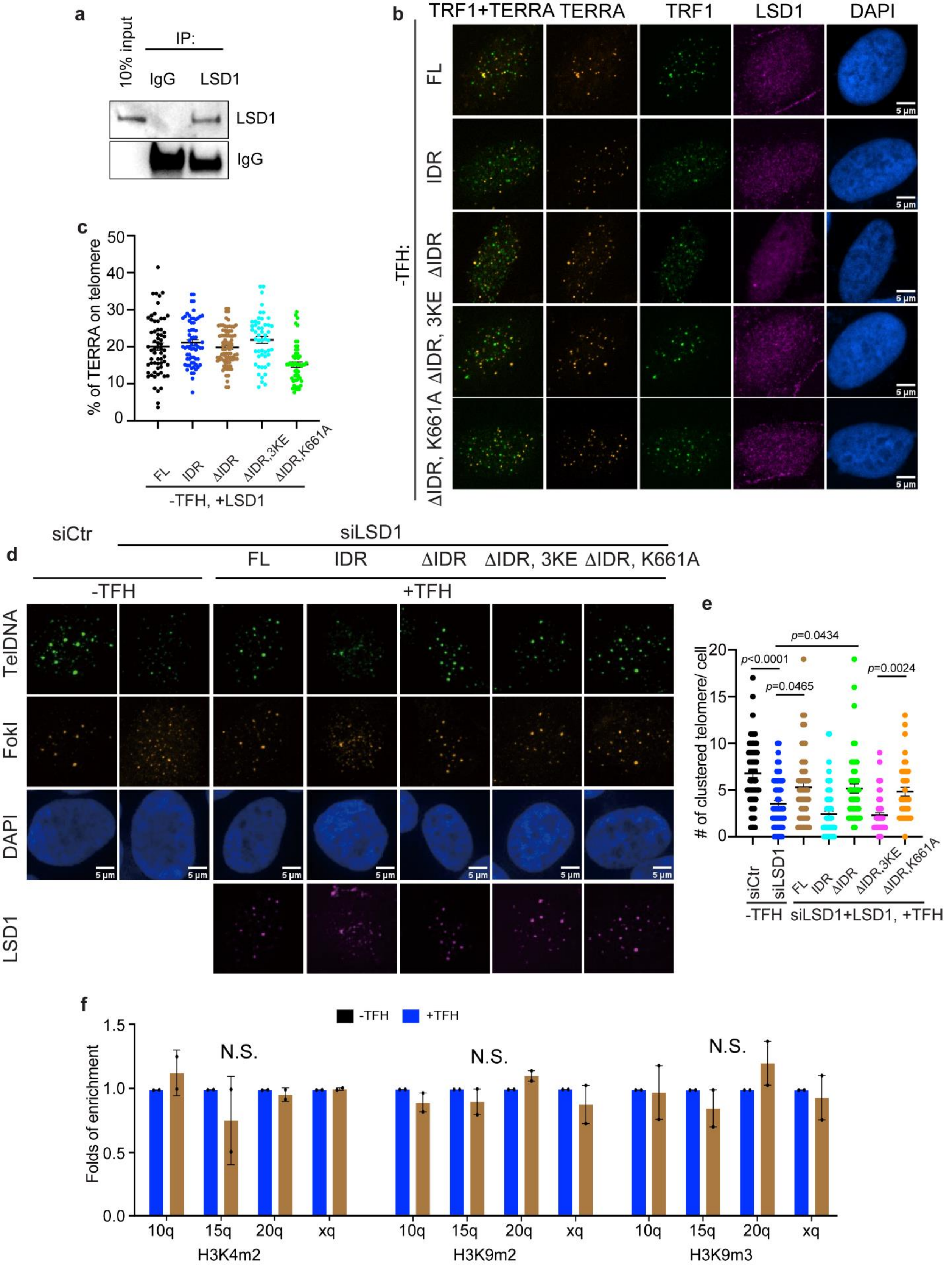
a
10% input
IP:
IgG
LSD1
LSD1
IgG
b
TRF1+TERRA
TERRA
TRF1
LSD1
DAPI
-TFH:
FL
IDR
ΔIDR
ΔIDR, 3KE
ΔIDR, K661A
5 μm
c
% of TERRA on telomere
FL
IDR
ΔIDR
ΔIDR,3KE
ΔIDR,K661A
-TFH, +LSD1
d
siCtr
siLSD1
FL
IDR
ΔIDR
ΔIDR, 3KE
ΔIDR, K661A
-TFH
+TFH
TelDNA
FokI
DAPI
LSD1
e
# of clustered telomere/ cell
p<0.0001
p=0.0465
p=0.0434
p=0.0024
siCtr
siLSD1
-TFH
siLSD1+LSD1, +TFH
f
-TFH
+TFH
Folds of enrichment
N.S.
10q
15q
20q
xq
H3K4m2
H3K9m2
H3K9m3

Supplementary Figure 4. LSD1 functional rescue requires nucleic acid binding but not enzymatic activity. a Western blotting was performed to examine immunoprecipitation (IP) efficiency of overexpressed LSD1 from U2OS cells in RNA-IP experiments. **b** Representative images and **c** quantification of TERRA (RNA FISH) on telomeres without adding dimerizer TFH for $LSD1^{FL}$, $LSD1^{IDR}$, $LSD1^{\Delta IDR}$, $LSD1^{\Delta IDR,\ 3KE}$, and $LSD1^{\Delta IDR,\ K661A}$ in Halo-TRF1 expressed U2OS cells (n= two independent experiments, one-way ANOVA). **d** Representative images and **e** quantification of telomere DNA FISH showing the rescue effect of telomere clustering after dimerization of $LSD1^{FL}$, $LSD1^{IDR}$, $LSD1^{\Delta IDR}$, $LSD1^{\Delta IDR,\ 3KE}$, and $LSD1^{\Delta IDR,\ K661A}$ to Halo-TRF1 in siLSD1 cells (n= two independent experiments, one-way ANOVA). **f** ChIP-qRCR assay was performed to detect telomeric $H3K4m^2$, $H3K9m^2$, and $H3K9m^3$ from indicated chromatin in Halo-TRF1 stable cells with or without adding TFH to induce eDHFR-LSD1 dimerization (n= two independent experiments, one-way ANOVA). Error bars are mean ± SEM. N.S., not significant. Source data are provided as a Source Data file.

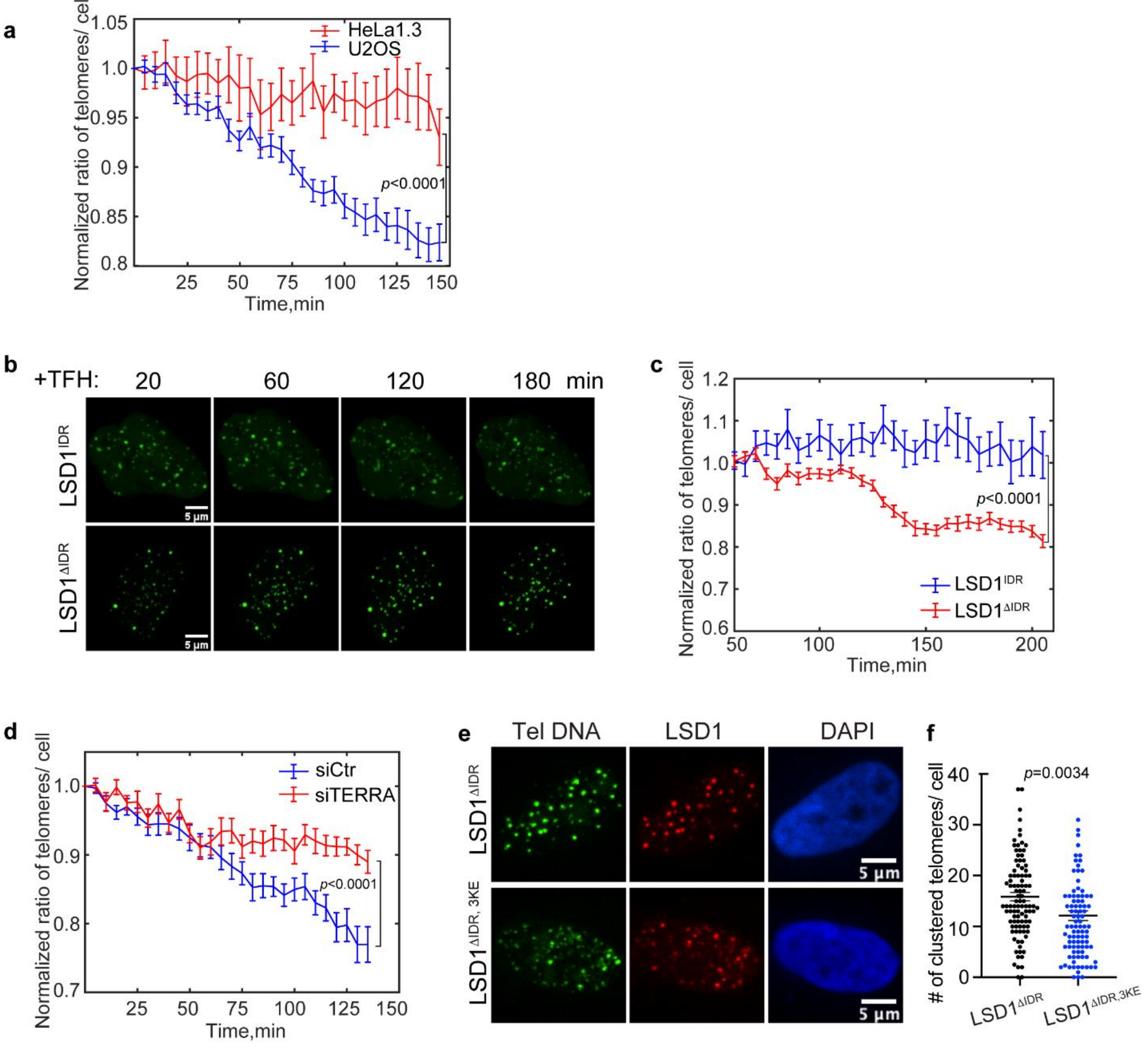

Supplementary Figure 5. Nucleic acid-binding site, but not IDR, is required for LSD1 phase separation. a Mean telomere number per cell after dimerizing mCh-eDHFR-LSD1 to Halo-GFP-TRF1 in Hela1.3 cells and U2OS cells (n=21 cells examined for U2OS and Hela1.3 over three independent experiments, two-tailed unpaired *t* test). **b** Representative images and **c** mean telomeres numbers per cell of U2OS cells expressing GFP-eDHFR-LSD1 (IDR and ∆IDR) and Halo-TRF1 after adding TFH to induce dimerization at indicated time points (n=13 cells examined for $LSD1^{IDR}$, n=12 cells examined for $LSD1^{\Delta IDR}$ over two independent experiments, two-way unpaired t test). **d** Mean telomere number per cell after dimerizing mCh-eDHFR-LSD1 to Halo-GFP-TRF1 with or without siTERRA (n=14 cells examined for siCtr, n=16 cells examined for siTERRA over three independent experiments, two-tailed unpaired *t* test). **e** Representative images and **f** quantification of telomere clustering (DNA FISH) after dimerizing mCh-eDHFR-LSD1 (∆IDR and ∆IDR, 3KE mutant) to Halo-TRF1 stably expressed in U2OS cells (n=110 cells examined for $LSD1^{\Delta IDR}$, n=102 cells examined for $LSD1^{\Delta IDR, 3KE}$ over three independent experiments, two-tailed unpaired *t* test). Source data are provided as a Source Data file.

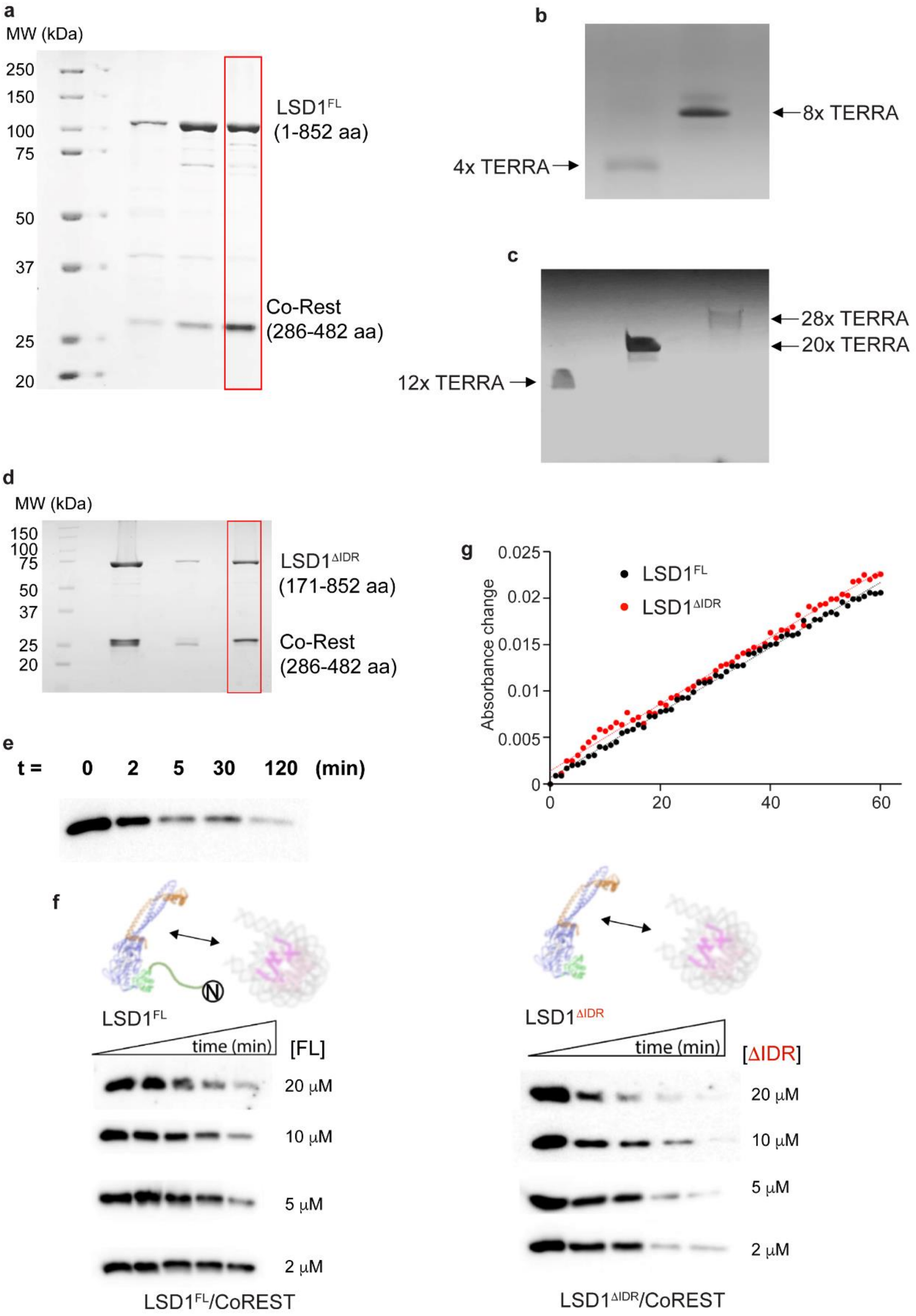
a
MW (kDa)
250
150
100
75
50
37
25
20
LSD1FL
(1-852 aa)
Co-Rest
(286-482 aa)
b
8x TERRA
4x TERRA
c
28x TERRA
20x TERRA
12x TERRA
d
MW (kDa)
150
100
75
50
37
25
20
LSD1ΔIDR
(171-852 aa)
Co-Rest
(286-482 aa)
g
Absorbance change
0.025
0.02
0.015
0.01
0.005
0
LSD1FL
LSD1ΔIDR
0
20
40
60
e
t = 0 2 5 30 120 (min)
f
N
LSD1FL
time (min)
[FL]
20 μM
10 μM
5 μM
2 μM
LSD1FL/CoREST
LSD1ΔIDR
time (min)
[ΔIDR]
20 μM
10 μM
5 μM
2 μM
LSD1ΔIDR/CoREST

Supplementary Figure 6. RNA purity of TERRA and demethylation activity by the purified LSD1/CoREST enzyme complexes used for *in vitro* phase separation assays. a Representative SDS-PAGE gels of full length $LSD1^{FL}$ in complex with CoREST (286-482). **b** Denaturing PAGE (8 M urea) of the 4X and 8X TERRA RNA constructs. **c** Denaturing PAGE (8 M urea) of the 12X, 20X, and 28X TERRA constructs and. All RNAs were folded in 10 mM Tris–HCl pH 7.4, 1 mM TCEP and 100 mM KCl buffer. **d** Representative SDS-PAGE gels of $LSD1^{\Delta IDR}$ (amino acids 171-852) in complex with CoREST (286-482). Purified LSD1-CoREST complexes (**a, d**) and purified RNAs (**b, c**) were used in enzyme activity studies, RNA binding, and phase separation-based assays. **e** Representative image of time course of LSD1 catalyzed demethylation of $H3K4me^2$ nucleosomes followed with anti $H3K4me^2$ antibody at time = 0, 2, 5, 30, and 120 minutes. **f** Representative demethylation time course reactions on $H3K4me^2$ nucleosome substrates, as previously established [95]. Western blot assays at identical concentrations and time intervals for either the full length LSD1 (black, 1-852) or truncated (red, 171-852) enzyme were performed, enabling a direct comparison of enzyme activity. **g** Time course reaction of LSD1 demethylation on $H3K4me^2$ peptide substrates, as measured indirectly through an established horseradish peroxidase (HRP) coupled assay that monitors H_2O_2 production under aerobic conditions, as previously established [95]. n= three independent experiments. Source data are provided as a Source Data file.

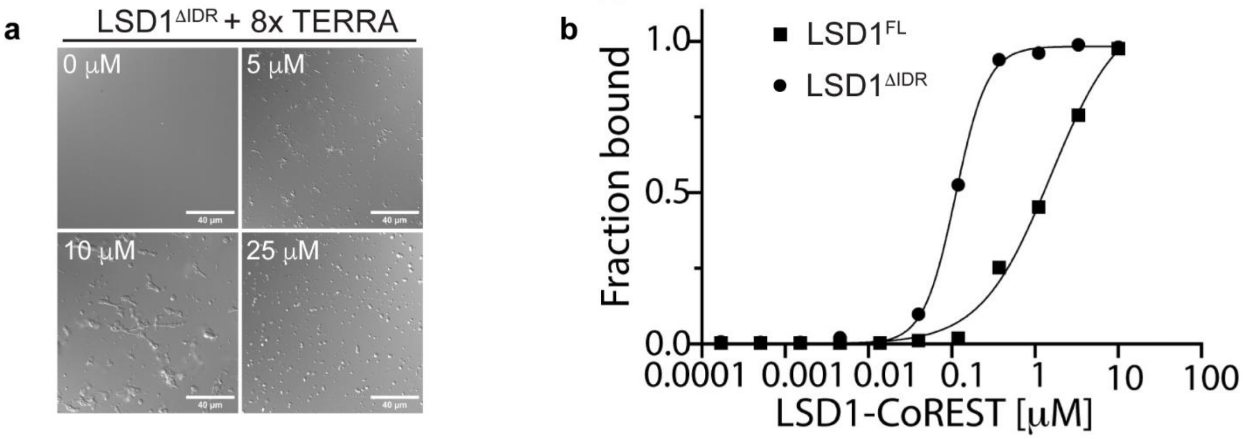

Supplementary Figure 7. Affinity of [UUAGGG]$_8$U RNA to the full length and truncated LSD1-CoREST. **a** Representative DIC images of truncated LSD1$^{\Delta IDR}$ with different amount of 8x TERRA. **b** Analysis of gel-mobility shift assay binding curves of (UUAGGG)$_8$U with truncated LSD1 (black circle, aa 171-852) and full length LSD1 (black squares, aa 1-852), where both complexes contain a truncated CoREST construct (aa 286-482). Plot shows the fraction of RNA bound (y-axis) at various LSD1-CoREST concentrations (log scale, x-axis), whereLSD1$^{\Delta IDR}$ and full length LSD1 bind RNA with apparent dissociation constants (K_d^{app}) of 112 +/- 7 nM and 1540 +/- 0.9 nM, respectively. n= three independent experiments. Source data are provided as a Source Data file.

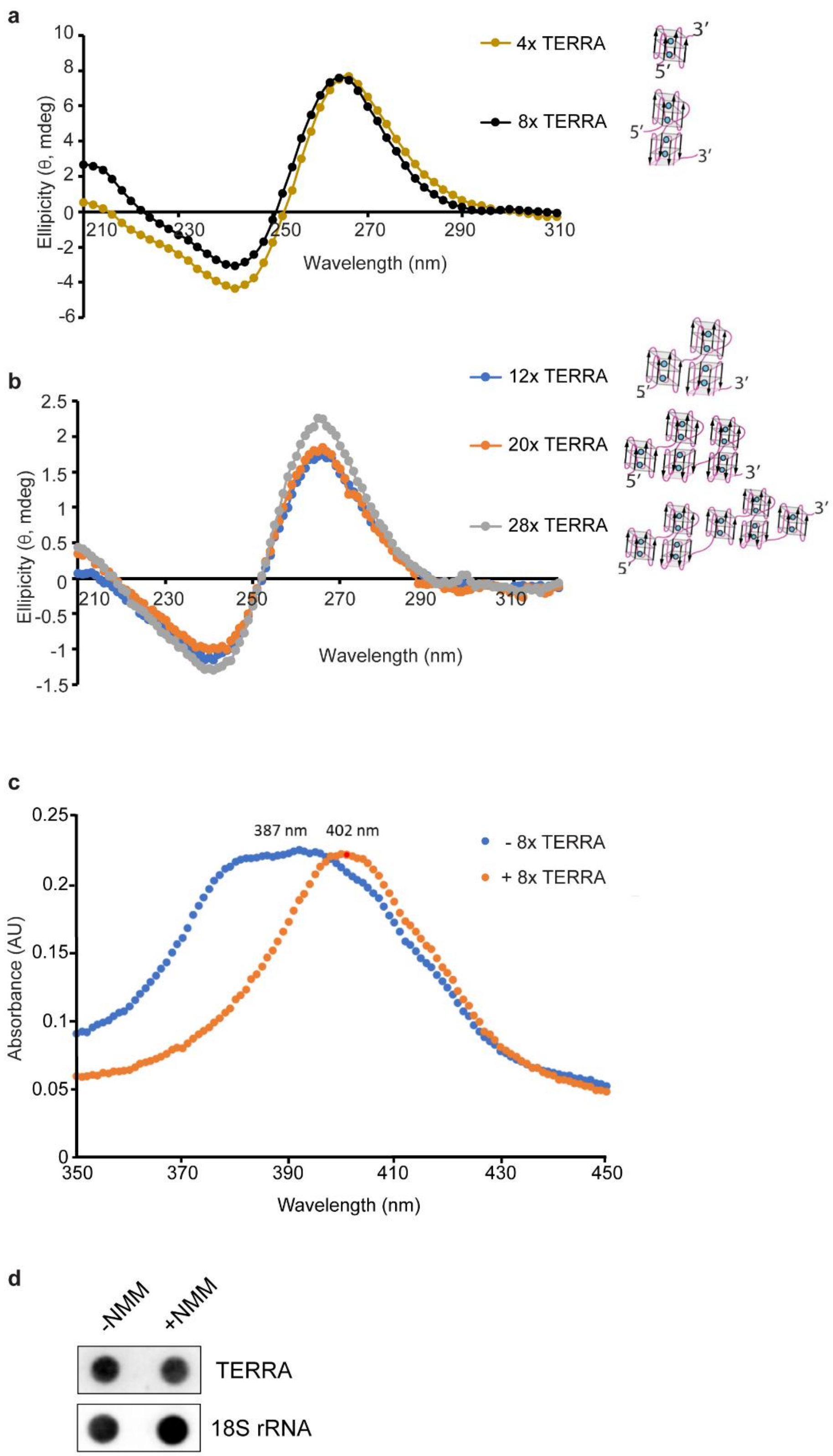
a
4x TERRA
8x TERRA
Ellipicity (θ, mdeg)
Wavelength (nm)
b
12x TERRA
20x TERRA
28x TERRA
Ellipicity (θ, mdeg)
Wavelength (nm)
c
387 nm
402 nm
- 8x TERRA
+ 8x TERRA
Absorbance (AU)
Wavelength (nm)
d
-NMM
+NMM
TERRA
18S rRNA

Supplementary Figure 8. Circular dichroism (CD) spectra of TERRA constructs and the effect of NMM in a 8X TERRA RNA binding. a-b Corresponding CD spectra of the (**a**) 4X, 8X, and (**b**) 12X, 20X, and 28X TERRA RNA constructs. CD spectra show that all RNAs fold into parallel-stranded guanosine-quadruplex conformations. All RNAs were folded in 10 mM Tris–HCl pH 7.4, 1 mM TCEP and 100 mM KCl buffer. Buffer for CD experiments contained 5 mM Na PO_4 (pH 7.4) and 10 mM KCl and was recorded at room temperature on a Chirascan^{TM} V100 CD spectrophotometer equipped with a 1 mm cell 1 nm band width. CD data were collected at 4 sec time per point with 0.1 ms timed intervals. Spectra from 200–320 nm were averaged over three scans, and the background was subtracted from a buffer matched sample. **c** The shift in the UV absorbance spectrum of NMM in the absence (blue, 387 nm) and presence of 8x TERRA RNA (orange, 402 nm) indicates that NMM binds the TERRA RNA used in our *in vitro* phase separation assays. In this spectrum, a 2:1 molar ratio of NMM:RNA provided an optimal spectral shift. **d** Northern dot-blot was used to detect total TERRA levels in U2OS cells treated with NMM for 24 hr. 18S rRNA serves as loading control. n= three independent experiments. Source data are provided as a Source Data file.

a

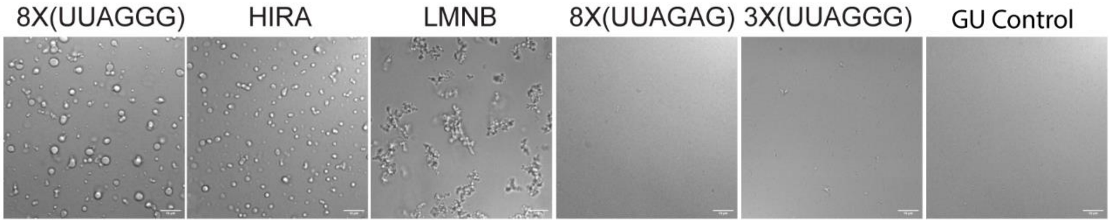

b

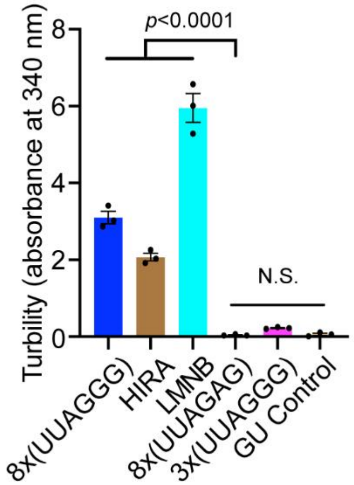

Supplementary Figure 9. LSD1 undergoes phase separation with different RNAs. **a-b** Representative DIC images **a** and turbidity assay **b** of LSD1$^{\Delta IDR}$ (50 μM) with the addition of different RNAs (50 μM). Turbidity measurements are shown as mean ± SEM (n= three independent experiments, one-way ANOVA). Source data are provided as a Source Data file.

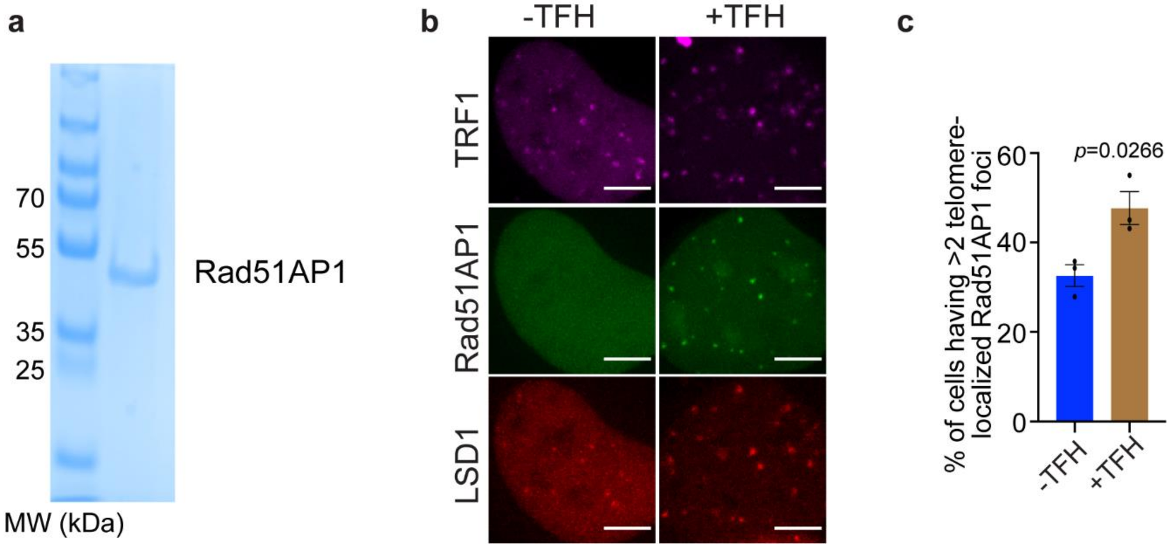

Supplementary Figure 10. LSD1 promotes Rad51AP1 localization at telomeres. a Coomassie blue gel to detect the purified RAD51AP1. **b** Representative images and **c** quantification of Rad51AP1 foci on telomeres after dimerization of LSD1$^{\Delta IDR}$ to Halo-TRF1 in U2OS cells (n= three independent experiments, two-tailed unpaired *t* test). Source data are provided as a Source Data file.

Supplementary Table1.
FRAP fitting results for TERRA in condensates containing 50 µM LSD1 and 50 µM 8x TERRA.

Droplet Bleached	R (µm)	T (sec)	D (μm^2/sec)	F_m
1	0.9	11.65	0.0174	0.6604
2	0.8	12.5	0.0128	0.6687
3	0.9	12.19	0.0166	0.7134
4	0.9	14.22	0.0142	0.5932
5	0.9	16.91	0.0120	0.6219
6	0.9	15.37	0.0132	0.6406
7	0.9	20.89	0.0097	0.6605
8	0.8	14.98	0.0107	0.5121
9	0.7	9.62	0.0127	0.4677
10	0.8	11.53	0.0139	0.5351
11	0.8	15.27	0.0105	0.5599
12	0.8	20.47	0.0078	0.5109
13	0.8	16.34	0.0098	0.5983
14	0.8	14.38	0.0111	0.5797
15	0.8	19.48	0.0082	0.5526
16	0.8	10.48	0.0153	0.5620
17	0.8	13.44	0.0119	0.5707
18	0.8	10.08	0.0159	0.5594
19	0.8	10.45	0.0153	0.5309
**Average:**			0.0126±0.0027	0.5841± 0.0635

Supplementary Table 2.

Oligos for siRNA, ChIP, biotinylated RNA, probes for dot blot, and non-G quadruplex RNAs

Name	Oligos
siLSD1_1	5'-GGAAGGCUCUUCUAGCAAU-3'
siLSD1_2	5'-GCAAGUGCUGUGAAAUAAC-3'
Control siRNA	5'-GGTTCATAAGAGGTGCCATGA-3'
siTERRA	5'-TAACCCTAACCCTAAC-3'
scramble control	5'-CACGTCTATACACCAC-3'
10q subtelomeric primers_F	5'-GCATTCCTAATGCACACATGAC-3'
10q subtelomeric primers_R	5'-TACCCGAACCTGAACCCTAA-3'
13q subtelomeric primers_F	5'-GCACTTGAACCCTGCAATACAG-3'
13q subtelomeric primers_R	5'-CCTGCGCACCGAGATTCT-3'
15q subtelomeric primers_F	5'-GCTGCATTAAAGGGTCCAGT-3'
15q subtelomeric primers_R	5'-AACCCTAACCACATGAGCAACG-3'
20q subtelomeric primers_F	5'-GAAGTTGCTGGGTTCTATGG -3'
20q subtelomeric primers_R	5'-ATGGTGCAGACACTGTGG-3'
xq subtelomeric primers_F	5'-CTTGAGCTCTCACCACTCAC-3'
xq subtelomeric primers_R	5'- GCCACGATAGCTTCTTCC -3'
GAPDH_F	5'-AGCCACATCGCTCAGACAC-3'
GAPDH_R	5'-GCCCAATACGACCAAATCC-3'
anti-FAM57B	5'-UUCCCCGUACACCCUCCCACCCUCCACC-3'
anti-MYO1B	5'-ACCCAGUGUUCCCCUCUUCCCUACCCACACCCA-3'
Poly U	5'-UUUUUUUUUUUUUUUUUUUUUUUUUUUUUUU-3'
52-Bio-TERRA	5'-UUAGGGUUAGGGUUAGGGUUAGGGUUAGGGUUAGGG UUAGGGUUAGGG-3'
52-Bio-Scramble	5'-UAAGACUAGCGCAUGAGUGCUCGUACUAGAGGUAGU CCUAGGUAGUC-3'
8x(UUAGAG)	5'UUAGAGUUAGAGUUAGAGUUAGAGUUAGAGUUAGAGU UAGAGUUAGAG-3'
LMNB RNA	5'-AGGGUGGGGGAGGGAGGUGGAGGGAGGGAAGGGU3'
HIRA RNA	5'-GGGGGAAUGGGGCAAAGGGGUGGGCGA-3'
GU RNA	5'-UGAAGGGUUUUUGUGUCUCUAUUUCCUUC-3'
TERRA probe for dot blot	5'-/BiotinTEG/TAACCC TAACCC TAACCC TAACCC TAACCC TAACCC TAACCC-3'
18S probe for dot blot	5'-/BiotinTEG/CGGAACTACGACGGTATCTG-3'
