# Supplementary material for: TERRA-LSD1 phase separation promotes R-loop formation for telomere maintenance in ALT cancer cells
Source: Nat Commun. 2024 Mar 9;15:2165. doi: 10.1038/s41467-024-46509-z (PMC10925046; doi:10.1038/s41467-024-46509-z)
Supplement: Supplementary file 3 — Description of Additional Supplementary Files [file 41467_2024_46509_MOESM3_ESM.pdf]

Description of Additional Supplementary Files

File Name: Supplementary Movie 1
Description: Recruiting LSD1$^{\Delta IDR}$ to telomeres without siTERRA. Movie for Fig. 4b. Left: composite of LSD1$^{\Delta IDR}$ (red) and TRF1 (green), middle: mCh-eDHFR-LSD1, right: Halo-GFP-TRF1. TFH was added to cells after the first time point to induce dimerization.

File Name: Supplementary Movie 2
Description: Recruiting LSD1$^{\Delta IDR}$ to telomeres in HeLa1.3 cells. Movie for Supplementary Fig. 5f. Left: composite of LSD1$^{\Delta IDR}$ (red) and TRF1 (green), middle: mCh-eDHFR-LSD1, right: Halo-GFP-TRF1. TFH was added to cells after the first time point to induce dimerization.

File Name: Supplementary Movie 3
Description: Recruiting GFP-eDHFR-LSD1^{IDR} to telomeres. Halo-TRF1 is co-transfected. Movie for Supplementary Fig. 5a. TFH was added to cells after the first time point to induce dimerization.

File Name: Supplementary Movie 4
Description: Recruit GFP-eDHFR-LSD1$^{\Delta IDR}$ to telomeres. Halo-TRF1 is transfected. Movie for Supplementary Fig. 5a. TFH was added to cells after the first time point to induce dimerization.

File Name: Supplementary Movie 5
Description: Recruiting LSD1$^{\Delta IDR}$ to telomeres with siTERRA (100 nM, 24 hr). Movie for Fig. 4b. Left: composite of LSD1$^{\Delta IDR}$ (red) and TRF1 (green), middle: mCh-eDHFR-LSD1$^{\Delta IDR}$, right: Halo-GFP-TRF1. TFH was added to cells after the first time point to induce dimerization.

File Name: Supplementary Movie 6
Description: Recruiting LSD1$^{\Delta IDR, 3KE}$ to telomeres. Movie for Fig. 4c. Left: composite of LSD1 (red) and TRF1 (green), middle: mCh-eDHFR-LSD1$^{\Delta IDR, 3KE}$, right: Halo-GFP-TRF1. TFH was added to cells after the first time point to induce dimerization.
