# Supplementary material for: TERRA-LSD1 phase separation promotes R-loop formation for telomere maintenance in ALT cancer cells
Source: Nat Commun. 2024 Mar 9;15:2165. doi: 10.1038/s41467-024-46509-z (PMC10925046; doi:10.1038/s41467-024-46509-z)
Supplement: Supplementary file 10 — Reporting Summary [file 41467_2024_46509_MOESM10_ESM.pdf]

nature portfolio

Corresponding author(s): Huaiying Zhang

Last updated by author(s): Feb 1, 2024

Reporting Summary

Nature Portfolio wishes to improve the reproducibility of the work that we publish. This form provides structure for consistency and transparency in reporting. For further information on Nature Portfolio policies, see our Editorial Policies and the Editorial Policy Checklist.

Statistics

For all statistical analyses, confirm that the following items are present in the figure legend, table legend, main text, or Methods section.

n/a	Confirmed	
☐	☒	The exact sample size (n) for each experimental group/condition, given as a discrete number and unit of measurement
☐	☒	A statement on whether measurements were taken from distinct samples or whether the same sample was measured repeatedly
☐	☒	The statistical test(s) used AND whether they are one- or two-sided<br>*Only common tests should be described solely by name; describe more complex techniques in the Methods section.*
☒	☐	A description of all covariates tested
☐	☒	A description of any assumptions or corrections, such as tests of normality and adjustment for multiple comparisons
☐	☒	A full description of the statistical parameters including central tendency (e.g. means) or other basic estimates (e.g. regression coefficient) AND variation (e.g. standard deviation) or associated estimates of uncertainty (e.g. confidence intervals)
☒	☐	For null hypothesis testing, the test statistic (e.g. F, t, r) with confidence intervals, effect sizes, degrees of freedom and P value noted<br>*Give P values as exact values whenever suitable.*
☒	☐	For Bayesian analysis, information on the choice of priors and Markov chain Monte Carlo settings
☒	☐	For hierarchical and complex designs, identification of the appropriate level for tests and full reporting of outcomes
☒	☐	Estimates of effect sizes (e.g. Cohen's d, Pearson's r), indicating how they were calculated

Our web collection on statistics for biologists contains articles on many of the points above.

Software and code

Policy information about availability of computer code

Data collection	-Western/Dot Blot: Azure Biosystems 300 (Chemiluminescent Western Blot Imager) and PhosphorImager<br>-qRT-PCR: The QuantStudio System 3 (Thermo Fisher A28567) with QuantStudio Analysis Software Version 1.5.2<br>-Imaging and Movie: Microscope ECLIPSE Ti2 (Nikon Instruments Inc.) and NIS Elements Version 5.30.01
Data analysis	-Imaging Plots: High-concentration images were preprocessed in NIS Elements Version 5.30.01. The final images were cropped and aligned in Adobe Illustrator 24.3.<br>-Western/Dot Blot: The original blots were preprocessed using imaging analysis software version 1.7.6.1202 and further cropped in ImageJ and aligned in Adobe Illustrator 24.3.<br>-Quantification Plots: High-concentration images were preprocessed and analyzed in NIS Elements Version 5.30.01. The raw data were subsequently processed using MATLAB R2020b. The final plots were generated using Prism 8.0. and model was generated in Bi<br>-qRT-PCR: The original data were analyzed using QuantStudio Analysis Software Version 1.5.2. The final plots were created using Prism 8.0.

For manuscripts utilizing custom algorithms or software that are central to the research but not yet described in published literature, software must be made available to editors and reviewers. We strongly encourage code deposition in a community repository (e.g. GitHub). See the Nature Portfolio guidelines for submitting code & software for further information.

Data

Policy information about availability of data

All manuscripts must include a data availability statement. This statement should provide the following information, where applicable:

- Accession codes, unique identifiers, or web links for publicly available datasets
- A description of any restrictions on data availability
- For clinical datasets or third party data, please ensure that the statement adheres to our policy

All data needed to evaluate the conclusions in the paper are present in the paper and the Supplementary Materials. Source data are provided with this paper. Important original data can be found at the 4DN data portal (https://data.4dnucleome.org/).

Research involving human participants, their data, or biological material

Policy information about studies with human participants or human data. See also policy information about sex, gender (identity/presentation), and sexual orientation and race, ethnicity and racism.

Reporting on sex and gender	n/a
Reporting on race, ethnicity, or other socially relevant groupings	n/a
Population characteristics	n/a
Recruitment	n/a
Ethics oversight	n/a

Note that full information on the approval of the study protocol must also be provided in the manuscript.

Field-specific reporting

Please select the one below that is the best fit for your research. If you are not sure, read the appropriate sections before making your selection.

☒ Life sciences ☐ Behavioural & social sciences ☐ Ecological, evolutionary & environmental sciences

For a reference copy of the document with all sections, see nature.com/documents/nr-reporting-summary-flat.pdf

Life sciences study design

All studies must disclose on these points even when the disclosure is negative.

Sample size	Sample size was chosen to balance replication and efficiency in the experiments. The exact sample size of the associated experiments was described in the source data. There was in general good correlation between replicates for the different experiments justifying the chosen sample size. No statistically calculation methods were used to predetermine sample size estimates. Sample size was determined based on the experimental results obtained from our preliminary experiments and published papers (doi: 10.1091/mbc.E19-10-0589; doi: 10.3389/fmolb.2021.785160)
Data exclusions	No data were excluded.
Replication	Number of replicates are described in the legends of the corresponding figures.
Randomization	For cell culture-based experiments, cells were split from the same batch of cells and randomly divided for each treatment in each replicate. Samples per replicate were processed together. Other experiments requiring randomization were not conducted.
Blinding	The investigators were not blinded to allocation during experiments and outcome assessment, as proper controls were already included during the experimental design. In these experiments, data analysis was strictly quantitative and were confirmed by multiple researchers who did not perform experiments. Therefore, blinding was not relevant in this study.

Behavioural & social sciences study design

All studies must disclose on these points even when the disclosure is negative.

Study description	*Briefly describe the study type including whether data are quantitative, qualitative, or mixed-methods (e.g. qualitative cross-sectional, quantitative experimental, mixed-methods case study).*

Research sample	*State the research sample (e.g. Harvard university undergraduates, villagers in rural India) and provide relevant demographic information (e.g. age, sex) and indicate whether the sample is representative. Provide a rationale for the study sample chosen. For studies involving existing datasets, please describe the dataset and source.*
Sampling strategy	*Describe the sampling procedure (e.g. random, snowball, stratified, convenience). Describe the statistical methods that were used to predetermine sample size OR if no sample-size calculation was performed, describe how sample sizes were chosen and provide a rationale for why these sample sizes are sufficient. For qualitative data, please indicate whether data saturation was considered, and what criteria were used to decide that no further sampling was needed.*
Data collection	*Provide details about the data collection procedure, including the instruments or devices used to record the data (e.g. pen and paper, computer, eye tracker, video or audio equipment) whether anyone was present besides the participant(s) and the researcher, and whether the researcher was blind to experimental condition and/or the study hypothesis during data collection.*
Timing	*Indicate the start and stop dates of data collection. If there is a gap between collection periods, state the dates for each sample cohort.*
Data exclusions	*If no data were excluded from the analyses, state so OR if data were excluded, provide the exact number of exclusions and the rationale behind them, indicating whether exclusion criteria were pre-established.*
Non-participation	*State how many participants dropped out/declined participation and the reason(s) given OR provide response rate OR state that no participants dropped out/declined participation.*
Randomization	*If participants were not allocated into experimental groups, state so OR describe how participants were allocated to groups, and if allocation was not random, describe how covariates were controlled.*

Ecological, evolutionary & environmental sciences study design

All studies must disclose on these points even when the disclosure is negative.

Study description	*Briefly describe the study. For quantitative data include treatment factors and interactions, design structure (e.g. factorial, nested, hierarchical), nature and number of experimental units and replicates.*
Research sample	*Describe the research sample (e.g. a group of tagged Passer domesticus, all Stenocereus thurberi within Organ Pipe Cactus National Monument), and provide a rationale for the sample choice. When relevant, describe the organism taxa, source, sex, age range and any manipulations. State what population the sample is meant to represent when applicable. For studies involving existing datasets, describe the data and its source.*
Sampling strategy	*Note the sampling procedure. Describe the statistical methods that were used to predetermine sample size OR if no sample-size calculation was performed, describe how sample sizes were chosen and provide a rationale for why these sample sizes are sufficient.*
Data collection	*Describe the data collection procedure, including who recorded the data and how.*
Timing and spatial scale	*Indicate the start and stop dates of data collection, noting the frequency and periodicity of sampling and providing a rationale for these choices. If there is a gap between collection periods, state the dates for each sample cohort. Specify the spatial scale from which the data are taken*
Data exclusions	*If no data were excluded from the analyses, state so OR if data were excluded, describe the exclusions and the rationale behind them, indicating whether exclusion criteria were pre-established.*
Reproducibility	*Describe the measures taken to verify the reproducibility of experimental findings. For each experiment, note whether any attempts to repeat the experiment failed OR state that all attempts to repeat the experiment were successful.*
Randomization	*Describe how samples/organisms/participants were allocated into groups. If allocation was not random, describe how covariates were controlled. If this is not relevant to your study, explain why.*
Blinding	*Describe the extent of blinding used during data acquisition and analysis. If blinding was not possible, describe why OR explain why blinding was not relevant to your study.*

Did the study involve field work? ☐ Yes ☐ No

Field work, collection and transport

Field conditions	*Describe the study conditions for field work, providing relevant parameters (e.g. temperature, rainfall).*
Location	*State the location of the sampling or experiment, providing relevant parameters (e.g. latitude and longitude, elevation, water depth).*
Access & import/export	*Describe the efforts you have made to access habitats and to collect and import/export your samples in a responsible manner and in*

Access & import/export	*compliance with local, national and international laws, noting any permits that were obtained (give the name of the issuing authority, the date of issue, and any identifying information).*
Disturbance	*Describe any disturbance caused by the study and how it was minimized.*

Reporting for specific materials, systems and methods

We require information from authors about some types of materials, experimental systems and methods used in many studies. Here, indicate whether each material, system or method listed is relevant to your study. If you are not sure if a list item applies to your research, read the appropriate section before selecting a response.

Materials & experimental systems

n/a	Involved in the study
☐	☒ Antibodies
☐	☒ Eukaryotic cell lines
☒	☐ Palaeontology and archaeology
☒	☐ Animals and other organisms
☒	☐ Clinical data
☒	☐ Dual use research of concern
☒	☐ Plants

Methods

n/a	Involved in the study
☒	☐ ChIP-seq
☒	☐ Flow cytometry
☒	☐ MRI-based neuroimaging

Antibodies

Antibodies used

Primary antibodies:

For Immunofluorescence
PML(PG-M3) Santa Cruz sc966, 1:50
TRF1, abcam, TRF78, ab10579, 1:100
TRF2, invitrogen, 4A794.15, MA1-41001, 1:200
GFP, thermo fisher A11122, 1:1000
mCherry, abcam ab183628, 1:1000
Flag, Cell signaling, D6W5B, #14793, 1:200
DHX36, Novus, NBP1-84286, 1:100
EZH2, Cell signaling, D2C9 XP, #5246, 1:200

For Westerb Blot
LSD1(AOF2)(C-ternimal), Sigma, L4418, 1:2000
Actin, abcam, mAbcam 8226, ab8226, 1:1000
hnRNP A1, (4B10), Novus, NB100-672, 1:1000
GFP, thermo fisher A11122, 1:1000
Flag, (D6W5B),Cell signaling, #14793, 1:1000

For Chromatin IP
Histone H3 (di methyl K9), abcam, mAbcam 1220, ab1220, 1:200
dimethyl-Histone H3 (Lys4), Millipore, 07-030, 1:100
Tri-Methyl-Histone H3 (Lys9) , (D4W1U), Cell signaling, #13969, 1:200
LSD1, Cell signaling, #2139, 1:1000
TRF2, invitrogen, 4A794.15, MA1-41001, 1:200
DNA-RNA Hybrid antibody, S9.6, Sigma, 1:100
mouse IgG Isotype Control, thermos, 10400C, 1:600

Second antibodies:
ALEXA FLUOR 488 anti-Rabbit, Thermos Fisher, A21206, 1:1000
ALEXA FLUOR 488 anti-mouse Thermos Fisher, A21202, 1:1000
ALEXA FLUOR 555 anti-rabbit Thermos Fisher, A21429, 1:1000
ALEXA FLUOR 555 anti-mouse Thermos Fisher, A31570, 1:1000
ALEXA FLUOR 647 anti-Rabbit Thermos Fisher, A31573, 1:1000
Anti-Rabbit IgG (H+L) Peroxidase Conjugated,Thermos Fisher,#31460, 1:10000
Anti-Mouse IgG (H+L) Peroxidase Conjugated,Thermos Fisher,#31430, 1:10000

Validation

All the antibodies used in this study were obtained from commercial sources and validated by the associated companies and indicated experiments in this study.
Second antibodies:
ALEXA FLUOR 488 anti-Rabbit, Thermos Fisher, A21206, https://www.thermofisher.com/antibody/product/Donkey-anti-Rabbit-IgG-H-L-Highly-Cross-Adsorbed-Secondary-Antibody-Polyclonal/A-21206
ALEXA FLUOR 488 anti-mouse Thermos Fisher, A21202, https://www.thermofisher.com/antibody/product/Donkey-anti-Mouse-IgG-H-L-Highly-Cross-Adsorbed-Secondary-Antibody-Polyclonal/A-21202
ALEXA FLUOR 555 anti-rabbit Thermos Fisher, A21429, https://www.thermofisher.com/antibody/product/Goat-anti-Rabbit-IgG-H-L-

Highly-Cross-Adsorbed-Secondary-Antibody-Polyclonal/A-21429
ALEXA FLUOR 555 anti-mouse Thermos Fisher, A31570, https://www.thermofisher.com/antibody/product/Donkey-anti-Mouse-IgG-H-L-Highly-Cross-Adsorbed-Secondary-Antibody-Polyclonal/A-31570
ALEXA FLUOR 647 anti-Rabbit Thermos Fisher, A31573, https://www.thermofisher.com/antibody/product/Donkey-anti-Rabbit-IgG-H-L-Highly-Cross-Adsorbed-Secondary-Antibody-Polyclonal/A-31573
Anti-Rabbit IgG (H+L) Peroxidase Conjugated,Thermos Fisher,#31460, https://www.thermofisher.com/antibody/product/Goat-anti-Rabbit-IgG-H-L-Secondary-Antibody-Polyclonal/31460
Anti-Mouse IgG (H+L) Peroxidase Conjugated,Thermos Fisher,#31430, https://www.thermofisher.com/antibody/product/Goat-anti-Mouse-IgG-H-L-Secondary-Antibody-Polyclonal/31430

Primary antibodies
Flag, Cell signaling, #14793, https://www.cellsignal.com/products/primary-antibodies/dykddddk-tag-d6w5b-rabbit-mab-binds-to-same-epitope-as-sigma-aldrich-anti-flag-m2-antibody/14793
TRF2, invitrogen MA1-41001, https://www.thermofisher.com/antibody/product/TRF2-Antibody-clone-4A794-15-Monoclonal/
TRF1, abcam ab10579, https://www.abcam.com/products/primary-antibodies/trf2--trf1-antibody-trf-78-ab10579.html
DNA-RNA Hybrid antibody, S9.6, Sigma, https://www.emdmillipore.com/US/en/product/Anti-DNA-RNA-Hybrid-Antibody-clone-S9.6,MM_NF-MABE1095?ReferrerURL=https%3A%2F%2Fwww.google.com%2F
PML(PG-M3) Santa Cruz sc966, https://www.scbt.com/p/pml-antibody-pg-m3
mCherry, abcam ab183628, https://www.abcam.com/products/primary-antibodies/mcherry-antibody-ab183628.html
GFP, thermo fisher A11122, https://www.thermofisher.com/antibody/product/GFP-Antibody-Polyclonal/A-11122
LSD1(AOF2)(C-ternimal), L4418,https://www.sigmaaldrich.com/US/en/product/sigma/l4418
hnRNPA1, Novus, NB100-672, https://www.novusbio.com/products/hnrnp-a1-antibody-4b10_nb100-672
Actin, abcam, ab8226, https://www.abcam.com/products/primary-antibodies/beta-actin-antibody-mabcam-8226-loading-control-ab8226.html
DHX36, Novus, NBP1-84286, https://www.novusbio.com/products/dhx36-antibody_nbp1-84286
EZH2, Cell signaling, D2C9, https://www.cellsignal.com/products/primary-antibodies/ezh2-d2c9-xp-174-rabbit-mab/5246
LSD1. Cell signaling, #2139,https://www.cellsignal.com/products/primary-antibodies/lsd1-antibody/2139
Histone H3 (di methyl K9), abcam, ab1220,https://www.abcam.com/products/primary-antibodies/histone-h3-di-methyl-k9-antibody-mabcam-1220-chip-grade-ab1220.html
dimethyl-Histone H3 (Lys4), Millipore, 07-030, https://www.emdmillipore.com/US/en/product/Anti-dimethyl-Histone-H3-Lys4-Antibody,MM_NF-07-030
mouse IgG, thermos, 10400C, https://www.thermofisher.com/antibody/product/Mouse-IgG-Isotype-Control/10400C

Eukaryotic cell lines

Policy information about cell lines and Sex and Gender in Research

Cell line source(s)	U2OS acceptor cells obtained from E.V. Makayev (Nanyang Technological University, Singapore), SaoS2 and SK-N-FI cells obtained from Samantha Pattenden (UNC-Chapel Hill, U.S.)
Authentication	Cell lines were not authenticated.
Mycoplasma contamination	All cell lines were tested negative for mycoplasma contamination on a regular basis.
Commonly misidentified lines (See ICLAC register)	No commonly misdentified cell line was used in this study.

Palaeontology and Archaeology

Specimen provenance	*Provide provenance information for specimens and describe permits that were obtained for the work (including the name of the issuing authority, the date of issue, and any identifying information). Permits should encompass collection and, where applicable, export.*
Specimen deposition	*Indicate where the specimens have been deposited to permit free access by other researchers.*
Dating methods	*If new dates are provided, describe how they were obtained (e.g. collection, storage, sample pretreatment and measurement), where they were obtained (i.e. lab name), the calibration program and the protocol for quality assurance OR state that no new dates are provided.*

☐ Tick this box to confirm that the raw and calibrated dates are available in the paper or in Supplementary Information.

Ethics oversight	*Identify the organization(s) that approved or provided guidance on the study protocol, OR state that no ethical approval or guidance was required and explain why not.*

Note that full information on the approval of the study protocol must also be provided in the manuscript.

Animals and other research organisms

Policy information about studies involving animals; ARRIVE guidelines recommended for reporting animal research, and Sex and Gender in Research

Laboratory animals	*For laboratory animals, report species, strain and age OR state that the study did not involve laboratory animals.*
Wild animals	*Provide details on animals observed in or captured in the field; report species and age where possible. Describe how animals were caught and transported and what happened to captive animals after the study (if killed, explain why and describe method; if released, say where and when) OR state that the study did not involve wild animals.*
Reporting on sex	*Indicate if findings apply to only one sex; describe whether sex was considered in study design, methods used for assigning sex. Provide data disaggregated for sex where this information has been collected in the source data as appropriate; provide overall numbers in this Reporting Summary. Please state if this information has not been collected. Report sex-based analyses where performed, justify reasons for lack of sex-based analysis.*
Field-collected samples	*For laboratory work with field-collected samples, describe all relevant parameters such as housing, maintenance, temperature, photoperiod and end-of-experiment protocol OR state that the study did not involve samples collected from the field.*
Ethics oversight	*Identify the organization(s) that approved or provided guidance on the study protocol, OR state that no ethical approval or guidance was required and explain why not.*

Note that full information on the approval of the study protocol must also be provided in the manuscript.

Clinical data

Policy information about clinical studies

All manuscripts should comply with the ICMJE guidelines for publication of clinical research and a completed CONSORT checklist must be included with all submissions.

Clinical trial registration	*Provide the trial registration number from ClinicalTrials.gov or an equivalent agency.*
Study protocol	*Note where the full trial protocol can be accessed OR if not available, explain why.*
Data collection	*Describe the settings and locales of data collection, noting the time periods of recruitment and data collection.*
Outcomes	*Describe how you pre-defined primary and secondary outcome measures and how you assessed these measures.*

Dual use research of concern

Policy information about dual use research of concern

Hazards

Could the accidental, deliberate or reckless misuse of agents or technologies generated in the work, or the application of information presented in the manuscript, pose a threat to:

No	Yes	
☐	☐	Public health
☐	☐	National security
☐	☐	Crops and/or livestock
☐	☐	Ecosystems
☐	☐	Any other significant area

Experiments of concern

Does the work involve any of these experiments of concern:

No	Yes	
☐	☐	Demonstrate how to render a vaccine ineffective
☐	☐	Confer resistance to therapeutically useful antibiotics or antiviral agents
☐	☐	Enhance the virulence of a pathogen or render a nonpathogen virulent
☐	☐	Increase transmissibility of a pathogen
☐	☐	Alter the host range of a pathogen
☐	☐	Enable evasion of diagnostic/detection modalities
☐	☐	Enable the weaponization of a biological agent or toxin
☐	☐	Any other potentially harmful combination of experiments and agents

Plants

Seed stocks	*Report on the source of all seed stocks or other plant material used. If applicable, state the seed stock centre and catalogue number. If plant specimens were collected from the field, describe the collection location, date and sampling procedures.*
Novel plant genotypes	*Describe the methods by which all novel plant genotypes were produced. This includes those generated by transgenic approaches, gene editing, chemical/radiation-based mutagenesis and hybridization. For transgenic lines, describe the transformation method, the number of independent lines analyzed and the generation upon which experiments were performed. For gene-edited lines, describe the editor used, the endogenous sequence targeted for editing, the targeting guide RNA sequence (if applicable) and how the editor was applied.*
Authentication	*Describe any authentication procedures for each seed stock used or novel genotype generated. Describe any experiments used to assess the effect of a mutation and, where applicable, how potential secondary effects (e.g. second site T-DNA insertions, mosiacism, off-target gene editing) were examined.*

ChIP-seq

Data deposition

☐ Confirm that both raw and final processed data have been deposited in a public database such as GEO.

☐ Confirm that you have deposited or provided access to graph files (e.g. BED files) for the called peaks.

Data access links<br>*May remain private before publication.*	*For "Initial submission" or "Revised version" documents, provide reviewer access links. For your "Final submission" document, provide a link to the deposited data.*
Files in database submission	*Provide a list of all files available in the database submission.*
Genome browser session<br>(e.g. UCSC)	*Provide a link to an anonymized genome browser session for "Initial submission" and "Revised version" documents only, to enable peer review. Write "no longer applicable" for "Final submission" documents.*

Methodology

Replicates	*Describe the experimental replicates, specifying number, type and replicate agreement.*
Sequencing depth	*Describe the sequencing depth for each experiment, providing the total number of reads, uniquely mapped reads, length of reads and whether they were paired- or single-end.*
Antibodies	*Describe the antibodies used for the ChIP-seq experiments; as applicable, provide supplier name, catalog number, clone name, and lot number.*
Peak calling parameters	*Specify the command line program and parameters used for read mapping and peak calling, including the ChIP, control and index files used.*
Data quality	*Describe the methods used to ensure data quality in full detail, including how many peaks are at FDR 5% and above 5-fold enrichment.*
Software	*Describe the software used to collect and analyze the ChIP-seq data. For custom code that has been deposited into a community repository, provide accession details.*

Flow Cytometry

Plots

Confirm that:

- [ ] The axis labels state the marker and fluorochrome used (e.g. CD4-FITC).
- [ ] The axis scales are clearly visible. Include numbers along axes only for bottom left plot of group (a 'group' is an analysis of identical markers).
- [ ] All plots are contour plots with outliers or pseudocolor plots.
- [ ] A numerical value for number of cells or percentage (with statistics) is provided.

Methodology

Sample preparation	*Describe the sample preparation, detailing the biological source of the cells and any tissue processing steps used.*
Instrument	*Identify the instrument used for data collection, specifying make and model number.*
Software	*Describe the software used to collect and analyze the flow cytometry data. For custom code that has been deposited into a community repository, provide accession details.*
Cell population abundance	*Describe the abundance of the relevant cell populations within post-sort fractions, providing details on the purity of the samples and how it was determined.*
Gating strategy	*Describe the gating strategy used for all relevant experiments, specifying the preliminary FSC/SSC gates of the starting cell population, indicating where boundaries between "positive" and "negative" staining cell populations are defined.*

- [ ] Tick this box to confirm that a figure exemplifying the gating strategy is provided in the Supplementary Information.

Magnetic resonance imaging

Experimental design

Design type	*Indicate task or resting state; event-related or block design.*
Design specifications	*Specify the number of blocks, trials or experimental units per session and/or subject, and specify the length of each trial or block (if trials are blocked) and interval between trials.*
Behavioral performance measures	*State number and/or type of variables recorded (e.g. correct button press, response time) and what statistics were used to establish that the subjects were performing the task as expected (e.g. mean, range, and/or standard deviation across subjects).*

Acquisition

Imaging type(s)	*Specify: functional, structural, diffusion, perfusion.*
Field strength	*Specify in Tesla*
Sequence & imaging parameters	*Specify the pulse sequence type (gradient echo, spin echo, etc.), imaging type (EPI, spiral, etc.), field of view, matrix size, slice thickness, orientation and TE/TR/flip angle.*
Area of acquisition	*State whether a whole brain scan was used OR define the area of acquisition, describing how the region was determined.*

Diffusion MRI ☐ Used ☐ Not used

Preprocessing

Preprocessing software	*Provide detail on software version and revision number and on specific parameters (model/functions, brain extraction, segmentation, smoothing kernel size, etc.).*
Normalization	*If data were normalized/standardized, describe the approach(es): specify linear or non-linear and define image types used for transformation OR indicate that data were not normalized and explain rationale for lack of normalization.*
Normalization template	*Describe the template used for normalization/transformation, specifying subject space or group standardized space (e.g. original Talairach, MNI305, ICBM152) OR indicate that the data were not normalized.*
Noise and artifact removal	*Describe your procedure(s) for artifact and structured noise removal, specifying motion parameters, tissue signals and physiological signals (heart rate, respiration).*

Volume censoring | *Define your software and/or method and criteria for volume censoring, and state the extent of such censoring.*

Statistical modeling & inference

Model type and settings | *Specify type (mass univariate, multivariate, RSA, predictive, etc.) and describe essential details of the model at the first and second levels (e.g. fixed, random or mixed effects; drift or auto-correlation).*

Effect(s) tested | *Define precise effect in terms of the task or stimulus conditions instead of psychological concepts and indicate whether ANOVA or factorial designs were used.*

Specify type of analysis: ☐ Whole brain ☐ ROI-based ☐ Both

Statistic type for inference | *Specify voxel-wise or cluster-wise and report all relevant parameters for cluster-wise methods.*

(See Eklund et al. 2016)

Correction | *Describe the type of correction and how it is obtained for multiple comparisons (e.g. FWE, FDR, permutation or Monte Carlo).*

Models & analysis

n/a	Involved in the study
☐	☐ Functional and/or effective connectivity
☐	☐ Graph analysis
☐	☐ Multivariate modeling or predictive analysis

Functional and/or effective connectivity | *Report the measures of dependence used and the model details (e.g. Pearson correlation, partial correlation, mutual information).*

Graph analysis | *Report the dependent variable and connectivity measure, specifying weighted graph or binarized graph, subject- or group-level, and the global and/or node summaries used (e.g. clustering coefficient, efficiency, etc.).*

Multivariate modeling and predictive analysis | *Specify independent variables, features extraction and dimension reduction, model, training and evaluation metrics.*
